# Awe weakens the AIDS-related stigma: The mediation effects of connectedness and empathy

**DOI:** 10.3389/fpsyt.2022.1043101

**Published:** 2022-12-02

**Authors:** Li Luo, Dong Yang, Yu Tian, Wei Gao, Jiemin Yang, Jiajin Yuan

**Affiliations:** ^1^The Affect Cognition and Regulation Laboratory (ACRLab), Faculty of Psychology, Southwest University, Chongqing, Institute of Brain and Psychological Sciences, Sichuan Normal University, Chengdu, China; ^2^School of Educational Science, Neijiang Normal University, Neijiang, China

**Keywords:** AIDS-related stigma, awe, connectedness, mediation, empathy

## Abstract

Stigma toward people with HIV or AIDS produces significant harms to their life and also hinders the prevention of AIDS. In the present study, we tested whether awe can weaken AIDS-related stigma and the mediating role of connectedness and empathy between them through a cross-sectional study (Study 1, *N* = 372) and two experimental studies (Study 2a and 2b, *N* = 110 and *N* = 180, respectively). Results showed that awe reduced AIDS-related stigma (Study 2a and 2b), *via* the serial mediation of connectedness and empathy (Study 1 and 2b). These findings suggest that the experience of awe increases one’s connectedness to the world, which then enhances empathy and decreases AIDS-related stigma. This study expands our understanding of the relationship between awe and stigma, providing empirical basis for decreasing social prejudice to others.

## Introduction

Stigma is defined as “an attribute that is deeply discrediting,” which lowers someone “from a whole and usual person to a tainted, discounted one” (1). Stigma appears when elements of stereotyping, labeling, status loss, separation, or discrimination occurs toward a specific person or a group of people (2). Thus, it is a derogatory and insulting label (3), which classifies stigmatized people as out-groups (2, 4).

It is known that AIDS poses a serious threat to people with it. However, the public stigma toward people with AIDS or HIV (later called HIV carriers, for short) is even more damaging to individuals than the disease itself (5). AIDS-related stigma is the attitude toward the carriers, including negative stereotypes (e.g., moral judgment), negative emotional reactions (e.g., anger and fear toward carriers), and discriminatory behaviors (e.g., treating carriers unfairly) (3). Stigma has many negative effects on physical and mental health of HIV carriers, who are ostracized, abandoned and restricted in various ways (6). For example, the Joint United Nations Program on AIDS (UNAIDS) pointed out that the HIV carriers infected with COVID-19 were not included in medical assistance. AIDS-related stigma is also a barrier to intervention of AIDS (7), because the carriers would conceal their infection and endanger healthy people. Therefore, it is necessary to examine the ways to mitigate social stigma toward HIV carriers.

Emotions can change the attitude and behavior toward stigmatized people (8). For example, disgust plays an important role in obesity stigma (9) and increases the rejection of a person with a physical deformity or disease (10). Compared to control groups, participants experienced authentic pride are more willing to help HIV carriers (11).

Awe refers to an emotion when we encounter vast and powerful stimuli that are beyond our understanding (12). Self-transcendent Emotion Theory proposes that awe is a self-transcendent emotion, which could encourage people to go beyond their own momentary desires and enhance the welfare of others (13, 14). In a recent netnographic study, Matson-Barkat et al. (15) analyzed the comments of the consumers of disability sport advertising. They found that those watching the disability sport advertising expressed much self-transcendent emotion (e.g., awe) toward the disabled athletes, which contributed to change their perception and attitude and lead to the destigmatization toward disability. Studies also found that those experiencing positive awe were more open and tolerant of others’ norm violations (16, 17). These findings provide support for the hypothesis that awe can decrease AIDS-related stigma.

If awe decreases AIDS-related stigma, what is the mechanism underlying this association? Feelings as Information Theory suggests that individuals usually take their feelings as a source of information which would affect the subsequent judgments and decisions (18). Connectedness is the main feeling produced by awe (19). Studies have found that awe reduces interpersonal psychological distance and promotes individuals to connect themselves with the world (14, 20). Yaden et al. (21) revealed that when astronauts were in the space, they felt the experience of awe. This experience produced an overview effect by broadening their boundaries, so that they no longer defined humanity by race or nation, but as a whole. Meanwhile, Brannon and Walton (22) found that connectedness to stigmatized groups increased participants’ interest in the culture and reduced stigma toward the target group. All of these are implied that awe may reduce stigma through the mediation effect of connectedness.

In addition, Broaden-and-Build Theory of Positive Emotions states that positive emotion shares the ability to broaden individuals’ momentary thought-action repertoires and build their enduring personal resources, which provides possibilities to support others (23). As a positive emotion (19, 24, 25), awe may increase empathy for others (26). Empathy, an other-oriented prosocial affect including compassion, sympathy, and tenderness (27), is an effective factor for performing kindness (28) and lessening stigma toward stigmatized groups (5, 29, 30). A recent study have showed that awe elicited by classical serotonergic psychedelic increases empathy (31), which means that experiencing awe benefits to the improvement of empathy. These evidences suggest that the mediation effect of empathy may be significant between awe and stigma. Studies also found that the sense of connectedness positively predicted empathy. Individuals perceiving a higher sense of connectedness are more empathic toward others’ sufferings (32). Similarly, clinical practice have proved that human-animal bond training enhances individuals’ empathy (33). Therefore, connectedness could promote empathy, and the two variables may mediate the weakening effects of awe on AIDS-related stigma serially.

AIDS-related Stigma is a widespread social phenomenon. Many studies have been done to reduce it (29), but little research has investigated how to reduce AIDS-related stigma from the perspective of emotions, especially self-transcendent emotions, such as awe. Therefore, the purpose of the study is to examine the impact of awe on AIDS-related stigma and the mechanism underlying the association. Across three studies, we investigated the weakening effect of awe on AIDS-related stigma (Study 1, 2a, and 2b) and the cognitive mechanism underlying the link from the perspective of connectedness and empathy (Study 1 and 2b). Study 1 is a cross-sectional study and Study 2a and 2b adopted an experimental approach. We hypothesized that awe may lead to lower AIDS-related stigma, and connectedness and empathy may mediate the association between them. The studies involving human participants were reviewed and approved by the internal ethics committee of Faculty of Psychology in Southwest University (Protocol number: H20081).

## Study 1

### Materials and methods

#### Participants

Study 1 was to test the association between dispositional awe and AIDS-related stigma, and the mediation roles of connectedness and empathy. We used the Monte Carlo Power Analysis for Indirect Effects application to determine the sample size (34). In previous research, it was found that awe had a moderately sized effect on prosocial and aggressive behavior (35). Therefore, we assume that correlations among the independent variable, mediators, and dependent variable are medium, *r* = 0.30 (*SD* = 0.10). At least 248 participants are needed to reach a power of 80. There were 372 college students from China voluntarily taking part in the survey online. The mean age of the participants was 20.35 ± 1.17 years and 206 were female.

#### Materials

##### Dispositional awe

The dispositional awe was assessed by the awe scale in Dispositional Positive Emotion Scale (DPES-awe) (36). This scale has six items. It is a seven-points Likert scale (1 = strongly disagree, 7 = strongly agree). The higher the score, the more often individuals experience awe. The Cronbach’s α was 0.90 in the present study.

##### Connectedness

Connectedness was assessed by The Inclusion of Other in the Self Scale, a pictorial scale measured through a choice of increasingly overlapping circles (31, 37). Participants responded on the scale to measure their connectedness felt to humanity and the world, respectively. The scores range from 1 (no overlap) to 7 (full overlap). The mean of the two items serves as the index of connectedness.

##### Empathy

Empathy Concern Scale (27) was used to assess the extent of empathy participants experienced for HIV carriers. The scale includes six adjective words, moved, warm, sympathetic, compassionate, tender, and softhearted. It is a seven-points scale (1 = not at all, 7 = extremely). The higher the score, the stronger the empathy that participants felt toward the carriers. The Cronbach’s α was 0.94 in the present study.

##### AIDS-related stigma

AIDS-related Stigma Questionnaire (38) was used to measure the stigma toward HIV carriers. This questionnaire includes three dimensions, that is, moral judgments (referring to individuals’ moral judgments toward the carriers), fear (referring to the fear of contracting AIDS through casual contact), and legal restriction (referring to the degree to which people agree with policies to restrict the carriers). There are 15 items. Participants respond to each item from 1 (strongly disagree) to 7 (strongly agree). The higher the score, the stronger the stigma toward the carriers. The Cronbach’s α was 0.71 in the present study.

#### Data analysis

Descriptive statistics were conducted in SPSS 23.0, and mediation analysis was performed in AMOS 23.0 (SPSS Inc., Chicago, IL, USA).

### Results

#### Descriptive statistics and correlations

The descriptive statistics and the Pearson’s correlations of the variables were shown in Table 1.

**TABLE 1:** Descriptive statistics and Pearson’s correlations for the variables.

Variables	*M* ± *SD*	1	2	3	4	5	6	7
Awe	5.54 ± 0.97	1						
Connectedness	4.69 ± 1.44	0.37***	1					
Empathetic concern	5.03 ± 1.16	0.50***	0.34***	1				
Moral judgment	2.19 ± 0.63	–0.22***	–0.22***	–0.35***	1			
Legal restriction	2.30 ± 0.93	–0.21***	–0.18***	–0.29***	0.48***	1		
Fear	2.54 ± 0.65	–0.20***	–0.21***	–0.34***	0.61***	0.49***	1	
Total stigma	2.40 ± 0.59	–0.24***	–0.24***	–0.39***	0.80***	0.74***	0.92***	1

***p < 0.001.

Results showed that awe was positively correlated with connectedness and empathy, and negatively correlated with AIDS-related stigma. Connectedness and empathy were negatively correlated with AIDS-related stigma.

#### Mediation effects analysis

**FIGURE 1:**
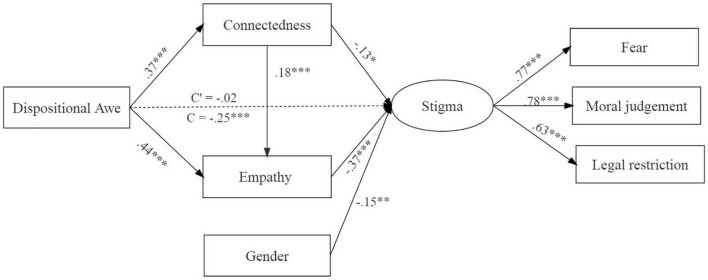
Mediation model for the effect of awe on AIDS-related stigma in Study 1. All path coefficients were standardized. The dotted line indicates the insignificant path coefficient. The model fitting was satisfactory, χ^2^ (10) = 11.72, *p* = 0.304, CFI = 1, NFI = 0.98, GFI = 0.99, RMSEM = 0.02. Dummy variable code: male = 1, female = 2. *p < 0.05, **p < 0.01, ***p < 0.001.

The Structural Equation Model and a parametric bootstrap procedure with 5,000 replications were used to calculate the 95% bias-corrected CIs for the indirect effects of the parameters and standard errors. The model (shown in Figure 1) included dispositional awe as the input variable, connectedness, and empathy as the mediators, AIDS-related stigma as the outcome variable, and gender as the control variable (39).

Results showed that the total effect of dispositional awe on AIDS-related stigma was significant (*p* < 0.001) and the direct effect was not (*p* = 0.836). Awe was associated with increased connectedness and empathy, *ps* < 0.001. There were significant indirect effects of awe on AIDS-related stigma through connectedness alone (mediation effect = –0.05, 95% CI [–0.10, –0.004], *p* = 0.031), empathy alone (mediation effect = –0.16, 95% CI [–0.23, –0.10], *p* < 0.001), and serial mediation of connectedness, and empathy (mediation effect = –0.02, 95% CI [–0.05, –0.01], *p* < 0.001). These results suggested that the weakening effect of awe on AIDS-related stigma was due to the strengthened sense of connectedness, which then enhanced individuals’ empathy and decreased stigma serially.

However, awe wasn’t manipulated in study 1 and we could not determine the causality between these variables. This issue was examined by Study 2a and 2b which used experimental approaches.

## Study 2a

### Materials and methods

#### Participants

We conducted *a priori* power analyses to calculate the required sample size, with effect size set at *d* = 0.50, power set at 0.80, and α = 0.05 (35). The necessary sample size was 102. There were 110 undergraduates (*N*_awe_ = 55; *N*_neutral_ = 55) who participated in the study (79 females). The participants’ mean age was 19.95 ± 1.17 years.

#### Materials

##### Emotional self-rating scale

Participants reported the extent to which they experienced each of four emotions (including amusement, awe, fear, and happiness) on a seven-points scale (1 = not at all, 7 = very strong) before and after emotional manipulation.

##### Emotional videos

This study induced emotion by watching videos. Participants in the awe group watched an awe-inspiring video describing natural scenery (1′59″), and those in the neutral group watched a traffic recorder video (4′27″).

##### AIDS-related stigma questionnaire

The AIDS-related stigma was measured the same as in Study 1, except that it measured the individual’s current attitude toward the HIV carriers.

#### Procedure

Before participants took part in the study, they read the informed consent, which stated there were two tasks in the experiment, including completing questionnaires and watching a video, and they were free to withdraw at any time and promised confidentiality of the data. After they confirmed these, the experiment began.

Participants first reported their current emotional feelings as the baseline and then were randomly assigned to awe or neutral group to watch videos to induce the target emotion. Next, they reported their emotions again as the manipulation check and completed the AIDS-related Stigma Questionnaire.

### Results

#### Manipulation check

We examined whether the emotional manipulation was effective. The pretest showed that there were no differences between awe and neutral group in amusement, awe, fear, and happiness, *ps* ≥ 0.573. The post-test showed that participants in the awe group experienced stronger feeling of awe (*M* = 5.41, *SD* = 1.32) than did those in the neutral group (*M* = 2.84, *SD* = 1.81), *t* (108) = 8.51, *p* < 0.001, *d* = 1.62. The post-test of awe for participants in the awe group was significantly higher than that of pretest (*M* = 2.44, *SD* = 1.25), *t* (54) = 14.28, *p* < 0.001, *d* = 1.93. These findings supported the success of emotional manipulation. For details, see Supplementary Table 1.

#### The effects of awe weakens AIDS-related stigma

By adopting ANOVA and including gender as the control variable, results showed that the effects of emotion on AIDS-related stigma were significant, *F* (107) = –4.49, *p* = 0.036, η^2^ = 0.04, but not on the sub-scales (*ps* > 0.057). Compared to neutral emotion (2.73 ± 0.33), the feeling of awe (2.56 ± 0.46) decreases the AIDS-related stigma. For details, see Supplementary Table 2.

## Study 2b

### Materials and methods

#### Participants

There were 182 college students who participated in the experiment. Two participants were excluded from data analysis as they did not follow the experimental procedures, leaving 180 valid cases (*N*_awe_ = 60; *N*_amusement_ = 60; *N*_neutral_ = 60; sample size adequate, determined by gpower). There were 108 females. The mean age was 20.26 ± 1.18 years.

#### Materials

##### Emotional self-rating scale

The emotional self-rating scale included seven emotions, anger, disgust, awe, sadness, fear, happiness, and amusement. Participants reported the extent to which they experienced each of the emotions on a seven-points scale (1 = not at all, 7 = very strong) before and after emotional manipulation.

The measures of connectedness, empathy for the HIV carriers, and AIDS-related stigma were assessed the same as those in Study 1, except that all of these represented how the participants were feeling at the moment.

Additionally, participants completed the dispositional questionnaires to ensure the homogeneity across the three groups, including dispositional awe assessed by DPES-awe (36), dispositional empathy assessed by Interpersonal Reactivity Index (40), prosocial tendencies assessed by Prosocial Tendencies Measure (41), and social desirability assessed by Marlowe–Crowne Social Desirability Scale (42). There were no differences in these variables among the three groups (*ps* > 0.077).

#### Procedure

The design and procedure in Study 2b were similar to those in Study 2a. The amusement group was chosen as the comparable group because both awe and amusement are positive emotions and can be elicited by an incongruity between one’s expectations and experience (26).

The participants completed the dispositional scales. Then, they reported their current emotional experiences as the baseline. Next, they completed the recalling-and-writing task to elicit the target emotion (26). Participants in the awe (or amusement) group were asked to recall and write about an experience in which they felt awe (or amusement), and then write down no less than ten sentences to describe it in detail. Participants in the neutral group were asked to recall and write a typical day’s routine. After completing the task, they reported their emotions again and the valence of the recalling (1 = very negative; 7 = very positive) as the manipulation check, and filled out the scales about connectedness, empathy, and AIDS-related stigma.

### Results

#### Manipulation check

MANOVA was applied to examine the success of the emotional manipulation. The pretest showed that there were no differences in the self-reported emotions among the three groups, *ps* ≥ 0.08. The post-test showed that participants in the awe group experienced stronger feelings of awe (*M* = 5.83, *SD* = 1.40) than did those in the amusement (*M* = 2.40, *SD* = 1.85) and neutral (*M* = 2.30, *SD* = 1.63) groups, *F* (2, 177) = 90.42, *p* < 0.001, η^2^ = 0.51. In turn, participants in the amusement group (*M* = 5.25, *SD* = 1.36) experienced a stronger feeling of amusement than did those in the awe (*M* = 1.40, *SD* = 1.17) and neutral (*M* = 2.37, *SD* = 1.65) groups, *F* (2, 177) = 121.87, *p* < 0.001, η^2^ = 0.58. Analysis for valence indicated no significant difference between awe (*M* = 5.70, *SD* = 1.39) and amusement (*M* = 5.40, *SD* = 1.26) conditions, *p* = 0.789, whereas both groups’ valence was more positive than that of the neutral group (*M* = 4.22, *SD* = 1.70; *ps* < 0.001; 95% mean differences CI [0.84, 2.13] for awe and [0.54, 1.83] for amusement group, respectively). These results suggested that emotional manipulation was successful in this study. For details, see Supplementary Table 3.

#### The influence of awe on connectedness, empathy, and AIDS-related stigma

**FIGURE 2:**
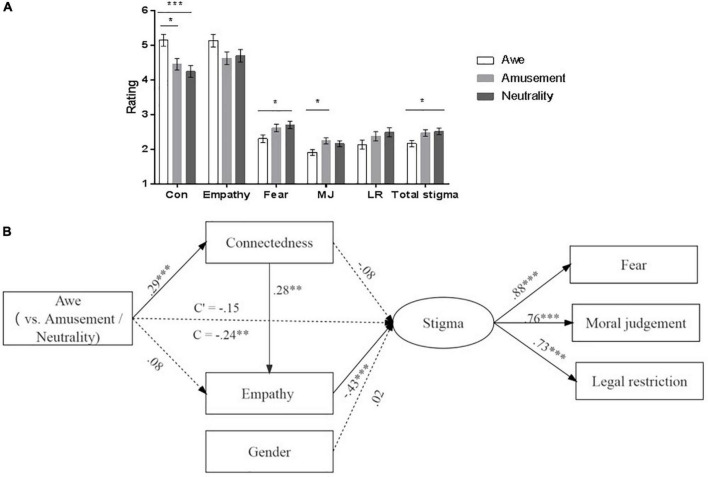
The effects of emotion on measures in Study 2b. **(A)** Shows the means and comparisons of measures as the function of emotion. Error bars represent standard errors. **(B)** Is the multiple mediation model from awe to AIDS-related stigma *via* connectedness and empathy. The model fitting was satisfactory, χ^2^ (10) = 15.79, *p* = 0.106, CFI = 0.98, NFI = 0.95, GFI = 0.98, RMSEM = 0.06. Con, connectedness; MJ, moral judgment; LR, legal restriction. Dummy variable code: awe group = 1, neutral group = amusement group = 0; male = 1, female = 2. *p < 0.05, **p < 0.01, ***p < 0.001.

MANOVA was used to test the effect of emotion on dependent variables and Bonferroni method was used for *post-hoc* pairwise comparisons. Gender was also controlled. The results, seeing Figure 2A, indicated a significant emotion effect on connectedness, moral judgment, fear, and total stigma, *ps* < 0.023, but not on empathy and legal restriction, *ps* > 0.106. *Post-hoc* pairwise comparisons showed that awe increased the sense of connectedness, and decreased moral judgment, fear and total stigma, compared with amusement, and neutral groups. Details were shown in Supplementary Table 4.

#### Mediation analysis

As indicated above, awe promoted the sense of connectedness and reduced the AIDS-related stigma. Pearson correlation analyses found that connectedness was associated with empathy (*r* = 0.31, *p* < 0.001) and AIDS-related stigma (*r* = –0.23, *p* = 0.002). So we conducted the mediation analysis by the same method as that in Study 1.

The results of mediation analysis indicated that the total effect from awe to AIDS-related stigma was significant (β = –0.24, 95% CI [–0.39, –0.08], *p* = 0.003), while the direct effect was not (β = –0.15, 95% CI [–0.30, 0.01], *p* = 0.075). The mediation effect of the serial mediation of connectedness and empathy (mediation effect = –0.04, 95% CI [–0.07, –0.02], *p* < 0.001) was significant. The mediation of connectedness and empathy alone was insignificant, *ps* > 0.223. These results suggested that awe reduced the AIDS-related stigma *via* the increased sense of connectedness and empathy serially (see Figure 2B).

## Discussion

Across three studies, we investigate the relationship between awe and AIDS-related stigma and the mediation effects of connectedness and empathy on this association. We found awe could decrease AIDS-related stigma, and this result was partially due to the fact that awe strengthened one’s sense of connectedness, which then enhanced empathy and consequently reduced stigma toward HIV carriers.

Previous studies found that self-transcendent emotions could reduce stigmatization toward athletes with disabilities (15), and the negative implicit and explicit prejudice toward gay men (8). In line with these studies, the present work used different approaches to confirm that the experience of awe lessened AIDS-related stigma. By contrast, we did not find the weakening effect of amusement on stigma. Self-transcendent emotions can bind individuals to the outside world, integrating themselves to a larger group (14). Meanwhile, awe has the element of “breaking set” in terms of beliefs about what is possible (43). Therefore, awe makes people more open and receptive to others (16). However, amusement can’t perform such a function and instead increases narcissism. Studies found that narcissism, characterized by grandiose fantasies and the need for admiration (44), together with reduced concern for others’ welfare and needs (45), increased stigma toward people with mental illness (46) and heightened the prejudice against out-group (47). Therefore, though amusement and awe share the same valence and a similar feature of other-oriented attention (48), they have different effects on stigma.

It was found that the association between awe and AIDS-related stigma was mediated by the serial mediation effects of connectedness and empathy (Study 1 and 2b). We often feel awe because the perceived object is vast (12), which makes us feel small and promotes us to integrate into a larger group (20, 26). Therefore, awe inspires us to connect ourselves with the outside world beyond the boundaries of self-identity (15). Previous studies found that the sense of connectedness changed the attitude and decision making toward others (22, 49). Additionally, van Mulukom et al. (31) observed a significant mediation model indicating that awe was associated with an increased feeling of connectedness, which then enhanced empathic drive and decreased narcissism. These evidences support the current mediation effects of connectedness and empathy between awe and AIDS-related stigma.

Different from Study 1, Study 2b did not find a mediation role of empathy between awe and AIDS-related stigma. Study 2b used two tasks to elicit state awe *via* experimental manipulation. While the feeling of awe measured in Study 1 resembles a trait-like feeling of awe, experimentally elicited awe in Study 2b was state-like. Some researchers consider empathy as an ability (50), an intellectual, higher-order or even effortful attribute (51), which may require long-term training (50). Evidence shows that elevated daily experience of awe is coupled with an increased level of empathy (31). Therefore, accidental awe experience may be not easy to enhance empathy instantly (52). This provides an explanation for the inconsistency between study 1 and 2b regarding the mediation of empathy.

Several limitations need to be illustrated. First, this study focused on reducing public stigma toward AIDS carriers. Personal experience with AIDS or with persons living with AIDS may be a potential influencing factor for AIDS-related stigma, though participants were randomly assigned to different emotional conditions. Therefore, this factor should be controlled for in future research. Meanwhile, that the influence of awe on carriers’ attitudes toward themselves is also worth investigating. Second, attitude may be explicit or implicit and we only measured the explicit AIDS-related stigma in this work. Whether the experience of awe reduces the implicit AIDS-related stigma needs further study. Third, awe can be triggered in many ways (53), and the present work only adopted the recalling-and-writing paradigm to examine the mechanism through which awe influences AIDS-related stigma. Future research could employ other ways to induce awe to verify the stability of the mediation role of connectedness and empathy between this link.

Taken together, the current findings reveal the role of awe in weakening AIDS-related stigma and its mechanisms. Awe can decrease stigma *via* enhancing individuals’ sense of connectedness with the outside world and empathy toward HIV carrier. The results suggested that enhancing the emotion of awe is a plausible method to change the negative attitude toward HIV carrier.

## Data availability statement

The original contributions presented in this study are included in the article/Supplementary material, further inquiries can be directed to the corresponding author.

## Ethics statement

The study was conducted in accordance with the Declaration of Helsinki, and approved by the 267 Internal Ethics Committee of the Faculty of Psychology in Southwest University (Protocol code H20081).

## Author contributions

LL and JMY conceived and designed the study. LL performed the experiments. LL and DY analyzed the data. LL, DY, JMY, and JJY wrote the manuscript. JMY and JJY contributed to funding acquisition. All authors contributed to the interpretation of the data and approved the final version of the manuscript.
