# Supplementary material for: Awe weakens the AIDS-related stigma: The mediation effects of connectedness and empathy
Source: Front Psychiatry. 2022 Dec 2;13:1043101. doi: 10.3389/fpsyt.2022.1043101 (PMC9755721; doi:10.3389/fpsyt.2022.1043101)
Supplement: Supplementary file 1 [file Data_Sheet_1.PDF]

Supplementary Materials

1 Study 2a (N = 110)

There were 110 undergraduates (N_{awe} = 55; $N_{neutral}$ = 55) randomly distributed to awe or neutral groups. Emotions were elicited by watching videos. Participants in the awe group watched an awe-inspiring video describing natural scenery, and those in the neutral group watched a traffic recorder video.

1.1 Manipulation check

Table S1 shows the descriptive statistics and comparison for pre- and post-test of emotions in study 2a. For pretest, there were no differences between awe and neutral group in amusement, awe, fear, and happiness, $ps \geq .573$. For post-test, results showed that the experiences of awe, amusement and happiness in awe group were higher than those in neutral group. And the experiences of amusement and happiness are lower than the experience of awe in awe group.

Table S1 Descriptive statistics and comparison for pre- and post-test of emotions

Measure	Awe	Neutrality	t	p
		Pretest of emotions		
amusement	1.75±.18	1.69±1.33	.26	.799
awe	2.44±1.25	2.35±1.72	.34	.735
fear	2.07±1.27	2.07±1.53	-.01	.995
happiness	1.96±1.26	1.82±1.33	.57	.573
		Post-test of emotions		
amusement	2.59±1.45	1.29±.81	5.80***	< .001
awe	5.41±1.32	2.84±1.81	8.51***	< .001
fear	2.29±1.36	2.02±1.48	.98	.328
happiness	1.54±.84	2.25±1.70	-2.79***	.006

1.2 The influence of awe on AIDS-related stigma

By adopting ANOVA and including gender as the control variable, results showed that the effects of emotion on AIDS-related stigma were significant, F (107) = -4.49, p = .036, η^2 = .04, but not on the sub-scales (ps > .057). Post hoc analyses, using Bonferroni method, showed that compared to neutral emotion (2.73±.33), the feeling of awe (2.56±.46) decreases the AIDS-related stigma. Table S2 shows the descriptive statistics and comparison for dependent variables as the function of emotions.

Table S2 Descriptive statistics and comparison for dependent variables as the function of emotions

Measure	Awe	Neutrality	*F*	*p*	*d*
Moral Judgement	2.54±.54	2.56±.48	.07	.797	.001
Legal restriction	2.06±.75	2.30±.75	2.81	.097	.03
Fear	2.72±.66	2.95±.52	3.71	.057	.03
Total stigma	2.56±.46	2.73±.33	4.49	.036	.04

2 Study 2b (N =180)

There were 182 college students participating in Study 2b. Two participants were excluded from data analysis as they did not follow the experimental procedures, leaving 180 valid cases (N_{awe} = 60; $N_{amusement}$ = 60; $N_{neutral}$ = 60). Participants were randomly distributed to one of the three groups.

The following results are based on the analysis of 180 valid participants in study 2b.

2.1 Manipulation check

For pretest, there were no differences between awe and neutral group in anger, awe, disgust, fear, amusement, sadness, and happiness, $ps \geq .08$.

The post-test showed that participants in the awe group experienced stronger feelings of awe than did those in the amusement and neutral groups, $F(2, 177) = 90.42$, $p < .001$, $\eta^2 = .51$. In turn, participants in the amusement group experienced a stronger feeling of amusement than did those in the awe and neutral groups, $F(2, 177) = 121.87$, $p < .001$, $\eta^2 = .58$.

Table S3 shows the descriptive statistics and comparison for pre- and post-test of emotions in study 2b.

Table S3 Descriptive statistics and analysis of variance (ANOVA) for pre- and post-test of emotions

Measure	Awe①	Amusement②	Neutrality③	ANOVA	Post-hoc statistics [95% CI mean difference]
Pretest of emotions					
Anger	2.12±1.56	1.78±1.30	1.88±1.45	F(2, 177) =.84, p =.43,η^2=.01	
Awe	3.40±1.90	2.70±1.61	2.92±1.68	F(2, 177) = 2.57，p = .08，η^2 = .028	
Disgust	1.87±1.32	1.77±1.20	1.75±1.19	F(2, 177) = .16，p = .856，η^2 = .002	
Fear	2.22±1.63	1.92±1.29	1.77±1.21	F(2, 177) = 1.63，p = .198，η^2 = .02	
Amusement	2.48±1.81	2.58±1.85	2.52±1.70	F(2, 177) = .05，p = .53，η^2 = .001	
Sadness	2.18±1.59	1.83±1.33	2.08±1.66	F(2, 177) = .83，p = .438，η^2 = .01	
Happiness	3.78±1.91	3.80±1.55	3.38±1.78	F(2, 177) = 1.09，p = .338，η^2 = .01	
Posttest of emotions					
Anger	1.93±1.78	1.67±1.17	1.83±1.21	F(2, 177) = .54, p = .582,η^2 = .01	
Awe	5.83±1.40	2.40±1.85	2.30±1.63	F(2, 177) = 90.42, p < .001,η^2 = .51	① - ② : p < .001***, [2.71, 4.16]<br>① - ③ : p < .001***, [2.81 , 4.26]
Disgust	1.33±.95	1.63±1.22	2.23±1.63	F(2, 177) =7.48，p = .001,η^2=.08	① - ③ : p = .001***, [-1.47, -.33]
Fear	2.17±1.62	1.73±1.35	1.78±1.22	F(2, 177) = 1.70; p = .185;η^2 = .02	
Amusement	1.40±1.17	5.25±1.36	2.37±1.65	F(2, 177) = 121.87; p < .001;η^2 = .58	① - ② : p < .001***, [- 4.47, -3.23]<br>① - ③ : p = .001***, [-1.59, -.35]<br>② - ③ : p < .001***, [2.26, 3.50]
Sadness	3.00±1.82	1.80±1.42	2.02±1.47	F(2, 177) = 9.81; p < .001;η^2 = .10	① - ② : p < .001***, [.50, 1.90]<br>① - ③ : p = .002**, [.29, 1.68]
Happiness	3.75±2.29	4.45±1.64	3.37±1.92	F(2, 177) = 4.67; p = .011;η^2 = .05	② - ③ : p < .033*, [.22, 1.95]

Note: Bonferroni method was used for post-hoc statistics. * p < .05; ** p < .01; ***p < .001.

2.2 The influence of awe on AIDS-related stigma

MANOVA was used to test the effect of emotion on dependent variables and Bonferroni method was used for post hoc pairwise comparisons. Gender was also controlled. The results, indicated a significant emotion effect on connectedness, F(2, 176) = 7.81, p = .001, η^2 = .08, moral judgment, F(2, 176) = 4.11, p = .018, η^2 = .05, fear, F(2, 176) = 3.86, p = .023, η^2 = .04, and total stigma F(2, 176) = 4.16, p = .017, η^2 = .05, but not on empathy and legal restriction, ps > .106. Post hoc pairwise comparisons showed that awe increased the sense of connectedness, and decreased moral judgment, fear and total stigma, compared with amusement and neutral groups.

Table S4 shows the descriptive statistics and comparison for pre- and post-test of emotions in study 2b.

Table S4 Descriptive statistics and Multivariate Analysis of Variance (MANOVA) for dependent variables as the function of emotions

Measure	Awe①	Amusement②	Neutrality③	ANOVA	Post-hoc statistics [95% CI mean difference]
Connectedness	5.14±1.39	4.45±1.19	4.25±1.30	$F(2, 176) = 7.81$, $p = .001$, $\eta^2 = .08$	① - ② : $p = .012$, [.12,1.27]<br>① - ③ : $p = .001$, [.3,1.47]
Empathy	5.13±1.45	4.62±1.29	4.69±1.47	$F(2, 176) = 2.26$, $p = .106$, $\eta^2 = .03$	
Fear	2.30±.81	2.62±.79	2.71±.90	$F(2, 176) =3.86$, $p = .023$, $\eta^2 = .04$	① - ③ : $p = .023$, [-.78, -.04]
Moral Judgement	1.91±.71	2.25±.64	2.16±.65	$F(2, 176) = 4.11$, $p = .018$, $\eta^2 = .05$	① - ② : $p = .019$, [-.63,-.04]
Legal restriction	2.14±1.02	2.38±.92	2.49±1.07	$F(2, 176) = 1.96$, $p = .445$, $\eta^2 = .02$	
Total stigma	2.17±.75	2.47±.67	2.52±.75	$F(2, 176) = 4.16$ $p = .017$, $\eta^2 = .05$	① - ③ : $p = .025$, [-.68, -.03]

3 Study 2b (N =182)

The following results are based on the analysis of 182 participants in study 2b.

Table S5 shows the descriptive statistics and comparison for pre- and post-test of emotions in study 2b. The results of table S4 and S5 show that the effects of emotion on connectedness, empathy and stigma are the same whether the two subjects were deleted or not.

Table S5 Descriptive statistics and Multivariate Analysis of Variance (MANOVA) for dependent variables as the function of emotions

Measure	Awe①	Amusement②	Neutrality③	ANOVA	Post-hoc statistics [95% CI mean difference]
Connectedness	5.14±1.37	4.45±1.19	4.25±1.30	F(2, 177) = 7.94, p < .001, η^2 = .08	① - ② : p = .011, [.12,1.25]<br>① - ③ : p = .001, [.32,1.45]
Empathy	5.14±1.44	4.63±1.29	4.69±1.47	F(2, 177) = 3.61 p =.092, η^2 = .04	
Fear	2.31±.80	2.62±.79	2.71±.90	F(2, 177) =3.96, p = .02, η^2 = .04	① - ③ : p = .024, [-.67, -.06]
Moral Judgement	1.93±.71	2.25±.64	2.16±.65	F(2, 177) = 3.64, p = .028, η^2 = .04	① - ② : p = .029, [-.61,-.02]
Legal restriction	2.18±1.03	2.38±.92	2.49±1.07	F(2, 177) = 1.45, p = .237, η^2 = .02	
Total stigma	2.18±.74	2.47±.67	2.52±.75	F(2, 177) = 3.86, p = .023, η^2 = .04	① - ③ : p = .034, [-.65, -.02]
